# The Irish Johne's Control Programme

**DOI:** 10.3389/fvets.2021.703843

**Published:** 2021-08-27

**Authors:** Lawrence Gavey, Lorna Citer, Simon J. More, David Graham

**Affiliations:** ^1^Animal Health Ireland, Carrick-On-Shannon, Ireland; ^2^Centre for Veterinary Epidemiology and Risk Analysis, UCD School of Veterinary Medicine, University College Dublin, Dublin, Ireland

**Keywords:** Johne's disease, paratuberculosis, control, programme, national, Ireland

## Abstract

The Irish Johne's Control Programme (IJCP) provides a long-term approach to the voluntary control of Johne's disease (JD) in Ireland, strongly supported by Irish cattle industry leadership. It leverages the establishment of Animal Health Ireland for control of animal diseases not regulated by the European Union. The IJCP has four objectives: facilitate protection against spread of JD to uninfected farms; reduce the level of infection when present; assure markets of JD control in Ireland; and improve calf health and farm biosecurity. Key IJCP elements are an annual veterinary risk assessment and management plan (VRAMP), annual whole herd test (WHT) by ELISA on blood or milk samples with ancillary faecal PCR testing of ELISA reactors, and Targeted Advisory Service on Animal Health (TASAH) investigations of infected herds. There are pathways for assurance of herds with continuing negative tests and for management of test-positive herds. Herdowners are responsible for on-farm activities, and specifically-trained (approved) veterinary practitioners have a pivotal role as technical advisors and service providers. The programme is supported by training of veterinarians, performance of testing in designated laboratories, documentation of policies and procedures, innovative data management for herd and test activities and for programme administration, training, and broad communication and awareness activities. Tools and systems are refined to address emerging issues and enhance the value of the programme. An Implementation Group comprising industry, government and technical leaders sets strategic direction and policy, advised by a Technical Working Group. Shared funding responsibilities are agreed by key stakeholders until 2022 to support herds in the programme to complete requirements. Herd registrations have increased steadily to exceed 1,800. National bulk tank milk surveillance is also being deployed to identify and recruit test-positive herds with the expectation that they have a relatively high proportion of seropositive animals. The programme will continue to innovate and improve to meet farmer and industry needs.

## Introduction

This case study describes the implementation of a control programme for Johne's disease (JD, paratuberculosis) in Ireland based on recommendations of Jordan et al. (1), some issues that arose during the initial stages of the programme, and ways in which these could be managed as the programme matures.

Agriculture is Ireland's oldest and largest industry. Agricultural exports, particularly dairy products, are economically and strategically important for the expanding Irish economy (2).

Ireland has a moist temperate climate. Irish dairy production is highly seasonal, aligned with seasonal pasture growth, to maximise production from natural inputs and the time spent by cows on pasture in a moist temperate climate. During winter, cows are generally housed to preserve soil health and are fed forage principally derived from harvesting surplus summer pasture growth. Milk production in January (winter) is <10% of peak production in May (spring). Ireland's dairy industry is structured on many small co-operatives as well as multinational agri-businesses, each of which independently determines farm milk prices.

Following abolition of the European Union quota system in 2015, the dairy industry has increased milk production by 40%. Ninety percent of dairy output is exported and the value of exports has grown from 2 to 5.2 billion in value since 2015 (3), further increasing its significance to the agri-food sector in Ireland.

Ireland has established an international reputation for reliable supply of agricultural and food products from pasture-based farm systems that are safe, of consistently high quality, and environmentally sustainable (2). However, markets are becoming increasingly competitive, and consumers are becoming more discerning about the products they purchase (4).

Stakeholders in the Irish dairy industry seek to enhance the reputation of existing products and to reassure customers through ongoing assessment of potential risks to which Irish food products could be exposed with an emphasis on preventative animal health measures (5).

Irrespective of whether *Mycobacterium avium* subspecies *paratuberculosis* (MAP; the aetiological agent for JD) is ever demonstrated to be a disease of zoonotic significance, trading success for Irish dairy products may be protected and enhanced by a demonstrable, effective and scientifically based programme to reduce the risk of presence of MAP. There is ongoing scientific investigation and research to determine the extent, if any, of an association between MAP and Crohn's disease in humans (6).

Although JD is considered to have a small impact on the productivity of the Irish dairy industry overall, individual affected herds may incur significant economic losses (7–9). JD reduces productivity through reduced milk yields, lower carcass value of affected milkers, costs of rearing more replacement animals and those that will be culled early, sub-clinical disease, and costs of diagnosis and treatments. It also negatively impacts animal welfare, antimicrobial use and greenhouse gas emissions (10). The economic impacts of Johne's infection are proportionate to prevalence and clinical and sub-clinical disease.

Clinical JD was first recorded in Ireland in 1920 in an imported cow. The prevalence of JD remained very low until the cessation of quarantine restrictions arising from introduction of the Single European Market which led to increased stock movements from mainland Europe after 1992, and significant importations of dairy cows to supply industry expansion particularly after quotas were lifted in 2015 (2, 11).

In 2009, as part of a long-term strategy for managing non-regulated diseases in the dairy industry, invited stakeholders participated in prioritising animal health issues for the newly formed entity, Animal Health Ireland (AHI). Using a process involving participatory consultation surveys and expert opinion, stakeholders ranked JD consistently as an important biosecurity risk disease requiring future management, even though the prevalence and production impacts of the disease were considered low at the time (12). Collectively a view was formed that the industry should proactively manage the potential for any emerging animal health risk and to continue to reassure markets by establishing a long-term Johne's control programme to mitigate this risk (12).

Ireland commenced a pilot voluntary Irish Johne's Control Programme in late 2013 to determine the feasibility of transitioning to a national programme. The pilot programme was based on the findings of a review (13) which identified herd level risk assessment, the practise of biocontainment and bioexclusion, and whole herd testing as the common bases of national programmes in six endemically infected countries (Australia, Canada, Denmark, the Netherlands, UK, USA). It also noted that repeated herd testing could improve detection of infection and increase levels of herd assurance.

Even now, relatively few countries have engaged in regional or national control programmes and only limited information is available on the effectiveness of those programmes in achieving their stated objectives (14).

To ensure the relevance and technical robustness of a future Irish Johne's Control Programme (IJCP), AHI commissioned an evaluation of testing strategies to determine the most appropriate approach (15) and a review of alternative surveillance methods for a national programme (16). A third paper (1) considered the elements required to effectively address the objectives of an Irish national control programme.

## Context

In Ireland, JD is notifiable to enable the Department of Agriculture, Food and the Marine (DAFM) to monitor the incidence, but there is no formal regulatory approach to control or eradication.

Vaccination against JD is not permitted, due to potential interference with testing for bovine tuberculosis (bTB).

The IJCP is a significant collaboration between the Irish dairy industry and DAFM, and is managed by AHI. AHI is an innovative not-for-profit partnership between farmers, agri-food businesses, private sector organisations and DAFM, that delivers programmes for non-regulated diseases of livestock (17). AHI provides a collegiate environment in which stakeholders collectively identify animal health issues, priorities and solutions. This model promotes shared responsibility for decision-making, funding and accountability for programme outcomes.

The IJCP has the support of all stakeholders involved in the programme, recognising it as having the capability to deliver a sustainable and internationally credible programme for the Irish dairy industry. Costs are shared by DAFM, the Rural Development Programme, individual milk processors and farmers. DAFM and milk processors have committed to maintain financial supports until at least the end of 2022.

The programme is advised by a Technical Working Group (TWG) comprising veterinary and technical personnel from private and government fields with interest or expertise in JD. This group ensures that the programme is evidence based and reflects contemporary scientific knowledge about JD control.

The programme is directed by an Implementation Group (IG) comprising AHI, DAFM, milk processors, farmer and veterinary representative organisations, milk recording organisations, breed societies, the Chair of the TWG and Animal Health and Welfare Northern Ireland (a sister not-for-profit organisation operating in Northern Ireland). This wide-ranging representation ensures that AHI stakeholders have a voice in the direction, design and implementation of the IJCP.

AHI takes advice from both the TWG and IG and has responsibility for the day-to-day management of the programme.

## Discussion

### About the IJCP

Prior to establishment of the IJCP, the herd-level true prevalence of JD on Irish dairy farms was estimated at 20% in 2005, based on the results of a serological survey (18), and was more recently estimated at 28% using a Bayesian methodology applied to 2013–2014 testing results limited to those herds participating in the IJCP (19).

The four objectives established by the IG for the IJCP are to:

- Enhance the ability of participating farmers to keep their herds clear of JD.
- Assist participating farmers to reduce the level of infection in their herds, where present.
- Provide additional reassurance to the marketplace in relation to Ireland's efforts to control JD.
- Improve calf health and farm biosecurity in participating farms.

To achieve these programme objectives, the following activities are required of participating herds:

- Annual herd level veterinary risk assessment and management plan (VRAMP; template available on request). The VRAMP is undertaken collaboratively by an approved veterinary practitioner (defined below) and farmer, to systematically review the bioexclusion and biocontainment risks of JD for the herd and agree on up to three management changes to reduce the likelihood of introduction and spread of MAP.
- Annual whole herd test (WHT) comprising ELISA screening tests with ancillary faecal culture or PCR testing of animals with positive or inconclusive ELISA results. The purposes of the whole herd test are either to increase herd-level assurance for test-negative herds or early detection and monitoring of progress towards infection control for test-positive herds. A WHT requires all bovine animals on the farm aged two years or more (‘eligible animals') to be tested by ELISA, using milk or blood samples.
- Ancillary testing is required for all animals with positive or inconclusive ELISA results unless the herd has a previous positive result for a faecal test. The purpose of the ancillary test is to confirm the presence of MAP in the herd.
- An epidemiological investigation follows the first confirmation of infection in a herd under a Targeted Advisory Service on Animal Health (TASAH) programme. The purpose is to identify the likely source and spread of infection and to inform VRAMP refinements.

The IJCP provides standardised protocols for testing and risk management, underpinned by training of veterinary practitioners and standards of laboratory testing to provide quality and consistency across the programme.

Private veterinary practitioners who provide essential support to herdowners under the programme must undertake specific training presented by AHI in the basic epidemiology of JD and programme operations. After completing training, an “approved veterinary practitioner” (AVP) may carry out VRAMPs, animal sampling and test interpretation. AVPs may also undertake a second tier of training to provide the TASAH epidemiological investigations.

All testing is conducted in designated laboratories, which are accredited to ISO 17025 for relevant tests, use only test kits approved by the Frederich-Loeffler-Institut with sensitivities and specificities indicated by the kit manufacturer, and participate in proficiency testing. Laboratories report results by electronic transfer to the programme database. DAFM provides National Reference Laboratory services.

The programme provides funded supports for activities. For all herds, costs of required ancillary PCR testing (for animal sampling by an AVP and laboratory testing) are fully funded by DAFM, and TASAH veterinary investigations are also fully funded. Ancillary faecal culture results are recognised by the programme, but this test is rarely used as it is not funded and requires a relatively long incubation period. For dairy herds that complete both the VRAMP and WHT annual requirements, DAFM funds the VRAMP and the milk processors fund herd testing assistance under agreed cost-sharing. Testing assistance is provided at the rate of EUR 2.75 per tested eligible animal for all herds in their first year. This rate is approximately the cost of ELISA testing using milk samples collected for milk quality and volume testing (milk recording). These supports are valued at EUR 550 for an average participating herd of 130 eligible animals. For testing of blood samples, there are additional costs for veterinary attendance, sampling and laboratory submission, borne by the herdowner. For test-negative herds, herd testing assistance declines over 3 years; for test-positive herds, assistance is maintained at the rate of EUR 2.75.

There is no compensation for culling test-positive animals as provided under some eradication programmes, since the IJCP only advises rather than requires such removals.

Completion of the WHT requires all animals to be sampled; a “sweeper” test of animals missed in a herd test (e.g., bulls, cull and sick animals, pre-calving heifers, and dry cows in the few year-round milking herds) is commonly required, usually using blood samples. Non-breeding animals which are held in an epidemiologically separate unit to the breeding herd may be exempted from testing.

The IJCP advises against ELISA testing within 90 days after tuberculosis skin testing, or within 7 days after calving (milk sample only), due to increased likelihood of false positive results.

Farmers and their advisers have access to a range of resources including the IJCP technical manual, user guides and standard operating procedures, forms and templates, monthly information bulletins and technical leaflets. The IJCP publishes an annual business plan with clearly articulated targets. While these elements that underpin the programme are not unique to the IJCP, and form the basis of control programmes in other developed countries, notably Canada (20), Germany (21) and England (22), we are unaware of any voluntary programme for non-regulated infectious diseases where the results from each of these activities have been fully integrated in a centralised database which also includes pedigrees and breeding history, movements and ultimate destinations of individual animals. This information may be accessed in real time by authorised users who are subject to the data sharing and privacy agreements which are in place.

Complementing the IJCP, DAFM undertakes animal disease surveillance including bulk tank milk testing (BTM) for a range of diseases, including JD. BTM may detect high-risk herds, so herds with positive BTM results are advised by a DAFM veterinary officer to join the IJCP, to avail of the funded tools to confirm infection and to control the spread and impacts of JD. However, BTM is considered a poor indicator of herd prevalence (16).

### Registration and Compliance

Farmer participation in the IJCP is supported and encouraged by key stakeholders including milk processors, milk recording organisations (MROs), DAFM and the veterinary profession.

At the end of 2020, there were 1,750 dairy herds registered in the IJCP, representing 11% of dairy herds and 18% of dairy cows in Ireland.

Six hundred thirty one herds continued from the pilot programme to Phase 1 of the IJCP. There were 301 new registrations in Phase 1 (late 2017–2018), 729 in 2019 and 139 in 2020 (Table 1). In consultation with herdowners, 40 inactive herd registrations were withdrawn in 2020.

**Table 1:** Summary of registered herds and testing results for the calendar years 2018–2020.

	**2017/8**	**2019**	**2020**
No. of nett new registrations	301	729	99
No. of registered herds (at year end)	939	1661	1760
No. of ELISA tests	141,657	206,486	215,963
No. ELISA positive or inconclusive	4,769	8,849	8,050
% ELISA positive or inconclusive	3.7	4.3	3.7
No. of ancillary tests	1,437	4,980	5,419
% ancillary positive	10.2	8.4	5.2
% animals with ELISA positive or inconclusive results that underwent required ancillary tests	30	56	67
No. herds conducting ancillary tests	311	802	947

The numbers of herds that completed both annual requirements of VRAMP and WHT were 1,376 (82%) in 2019 and 1,325 herds (75%) in 2020. In 2020, 326 registered herds (13%) were inactive and the remaining 99 herds either part-completed the WHT and/or did not complete the VRAMP.

There has been a substantial improvement in the number and proportion of animals and herds conducting the required ancillary PCR tests, from 30% in 2018 to 67% in 2020. Many animals requiring outstanding PCR tests are no longer available for testing. If required ancillary testing of an animal with a positive or inconclusive ELISA result is not conducted, the animal and its herd are considered by the programme to be infected despite the presence of JD not being confirmed, which may have adverse consequences for the herd's future assurance standing.

Registered herds self-selected into the programme and thus do not constitute a random sample of Irish herds, therefore extrapolation of programme prevalence data to the national herd is inappropriate. Additionally, due to the large number of herds (691) that have had at least one animal with a positive or inconclusive ELISA test result without an ancillary PCR test, the number or proportion of herds in the programme that are truly infected cannot be accurately calculated.

For the 2020 programme year (including an extension for completion of requirements until 31 January 2021-discussed later), there were 224,364 ELISA tests conducted, 105,642 (47%) on milk samples and 118,722 (53%) on blood. Of these ELISA tests, 8,466 (3.8%) results were positive or inconclusive (Table 2).

**Table 2:** Percent of positive and inconclusive individual ELISA test results per calendar year.

**Sample**	**Result**	**2018**	**2019**	**2020**
Blood	Positive	2.3	2.4	2.2
	Inconclusive	0.6	0.8	0.7
	Total (positive or inconclusive)	2.8	3.2	2.9
Milk	Positive	3.1	4.2	2.5
	Inconclusive	1.0	1.3	2.1
	Total (positive or inconclusive)	4.2	5.4	4.6

ELISA tests of milk samples have consistently higher rates of positive and inconclusive results than tests of blood samples (Table 2); however, a preliminary analysis shows the rate of PCR positive results is lower for animals ELISA-tested by milk than by blood. This suggests that ELISA testing of milk samples has a lower specificity than testing of blood samples, with this potentially further influenced by stage of lactation, age or seasonal conditions (23). The proportion of positive results for milk-ELISA tests was higher in 2019 than other years, spiking at the end of the 2019 lactation. These characteristics of milk testing are undergoing further analysis.

There were 5,419 ancillary tests in 2020, with positive results for 281 (5.1%) samples (not herds). Since 2018, the proportions of herds undertaking the required ancillary testing of animals following positive or inconclusive ELISA results have increased (Table 1) but the incidence of positive PCR test results has declined (10.2% in 2018, 8.4% in 2019, and 5.2% in 2020). At least in part, this declining incidence is due to the exclusion of known-infected herds from funded PCR testing, skewing testing towards herds that are not infected.

### Farmer Participation

Diminishing participation, for both recruiting new herds and completion of annual requirements by registered herds, is a current challenge. Multiple IG members, particularly milk processors, PVPs and MROs, reported that Brexit and COVID-19 concerns disrupted efforts to promote registration to their suppliers and clients for most of 2020. Farm access for sampling (both blood and milk) and VRAMPs was constrained by government restrictions, and later by aversion of farmers and their veterinary advisors to the risk of spreading COVID-19. The leadership of the milk processors in promoting the IJCP was re-directed towards ensuring the safety of suppliers and staff, continuity of supply and managing additional market risks created by Brexit and the COVID-19 pandemic.

The costs of the programme for participating farmers are low (or a minor cost if blood-sampling), compared to the benefits. However, the majority of dairy farmers in Ireland have yet to register in the programme.

This reticence is consistent with findings that “farmers are not solely influenced by economic consequences of management changes: (24). Smith and Findeis (25) note that innovations leading to the adoption of changed management practises may be challenging where the benefit arising from the changes may occur in the future, or where there may be no consequences for retaining the status quo, for example, in the IJCP context, where a herd remains uninfected with JD.

Similarly for mastitis research, Regan et al. (26) found that voluntary uptake of milk recording on Irish farms was influenced by perceived risk of a mastitis outbreak and argue that risk perception should be considered when promoting a behavioural change that may not provide instant feedback on its benefits. Within a voluntary programme such as the IJCP, the drivers for participation do not come from extrinsic pressures in the form of regulations, which can have benefits on meaningful engagement and participation. Instead, social and psychological factors play a part in increasing intrinsic motivation to enrol and continue engagement. Ritter et al. (27) highlight factors such as perceived risk, confidence in professional advice and discussion amongst peers as factors and reiterate the need for tailored communication strategies, while Sorge et al. (28) illustrate the need to consider the perceived zoonotic risk of JD and time resources when promoting farmer participation and engagement in a JD programme.

### Flexible Approach to Testing

ELISA testing of milk samples during lactation typically extends from April until October, whereas ELISA testing of blood samples collected by AVPs (for either the whole-herd or only sweeper tests) is concentrated towards the end of the calendar year, and due to logistical and seasonal consideration collection may be delayed, on occasion occurring in the first month of the following calendar year.

For efficiency, AVPs often carry out blood and faecal testing at the same time as conducting the annual bTB test and/or annual VRAMP. The blood and faecal sampling and VRAMP activities may be purposely delayed until the winter housing period, to minimise inconvenience and animal time-off-pasture for sampling and to coincide VRAMPs with the pre-calving period when the most effective interventions can be deployed.

Despite the financial incentive of testing assistance, many herds have been unable to complete the annual WHT and VRAMP requirements before the end of the calendar year, necessitating one-month extensions of the programme years. Thirty percent of herds utilised this extension to meet the programme requirements for the 2020 programme year. It is proposed to integrate this flexibility to the programme in future years, notwithstanding the financial and administrative complexities.

### Information Management and Communications

Testing and VRAMP data are held in the Irish Cattle Breeding Federation (ICBF) database. Designated laboratories upload test results for the IJCP, and AVPs upload VRAMP and TASAH investigation reports. The database also contains genetic and production information for all registered cattle in Ireland, including animal birth dates, pedigrees, livestock movements, disposal of animals, and bTB test dates (but not results) to assist interpretation of JD ELISA test results.

**Figure 1:**
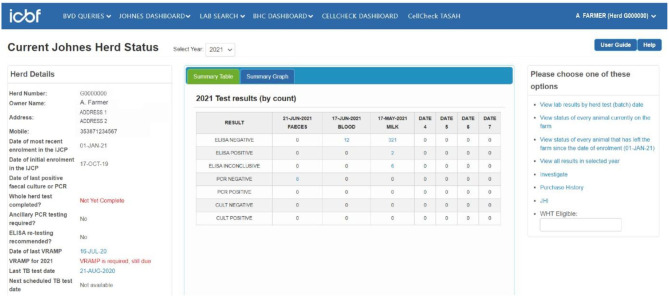
Johne's herd-level dashboard landing page.

**Figure 2:**
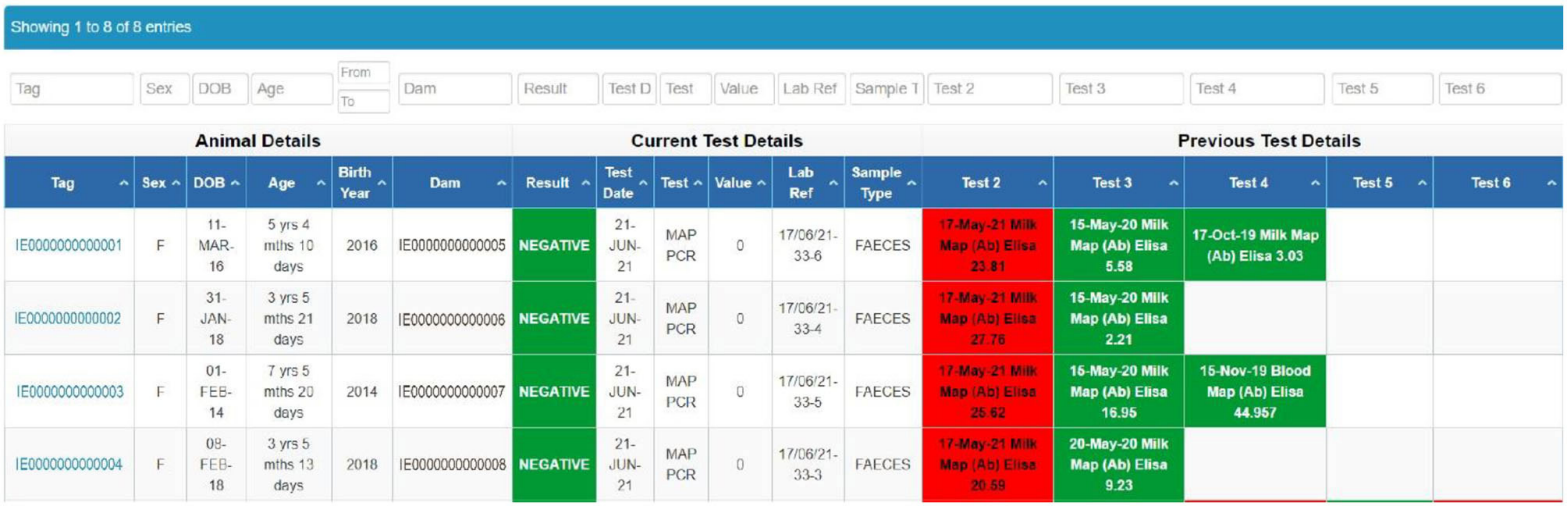
ICBF display of listed animals.

Herdowners and AVPs can readily access the database via computer or mobile devices. The primary ICBF screen (“herd-level dashboard,” Figure 1) displays test results, the date of the most recent VRAMP and specifically highlights outstanding required activities. Other screens provide filterable and sortable animal level details, including their dates of birth, age, sex, dam and a colour-coded test history (Figure 2), and further details of animals. A reporting tool enables download of this information in both Excel® and “pdf” format to enable further herd and animal analyses. The integration of data, from the Johne's programme with that of animal and herd productivity and management, facilitates interpretation of results, evidence-based decision-making, and monitoring of progress.

The IJCP provides herdowners and AVPs with access to information about JD, the programme and financial supports available at the time. The principal but not exclusive point of contact for this information is the AHI website, www.animalhealthireland.ie.

**Figure 3:**
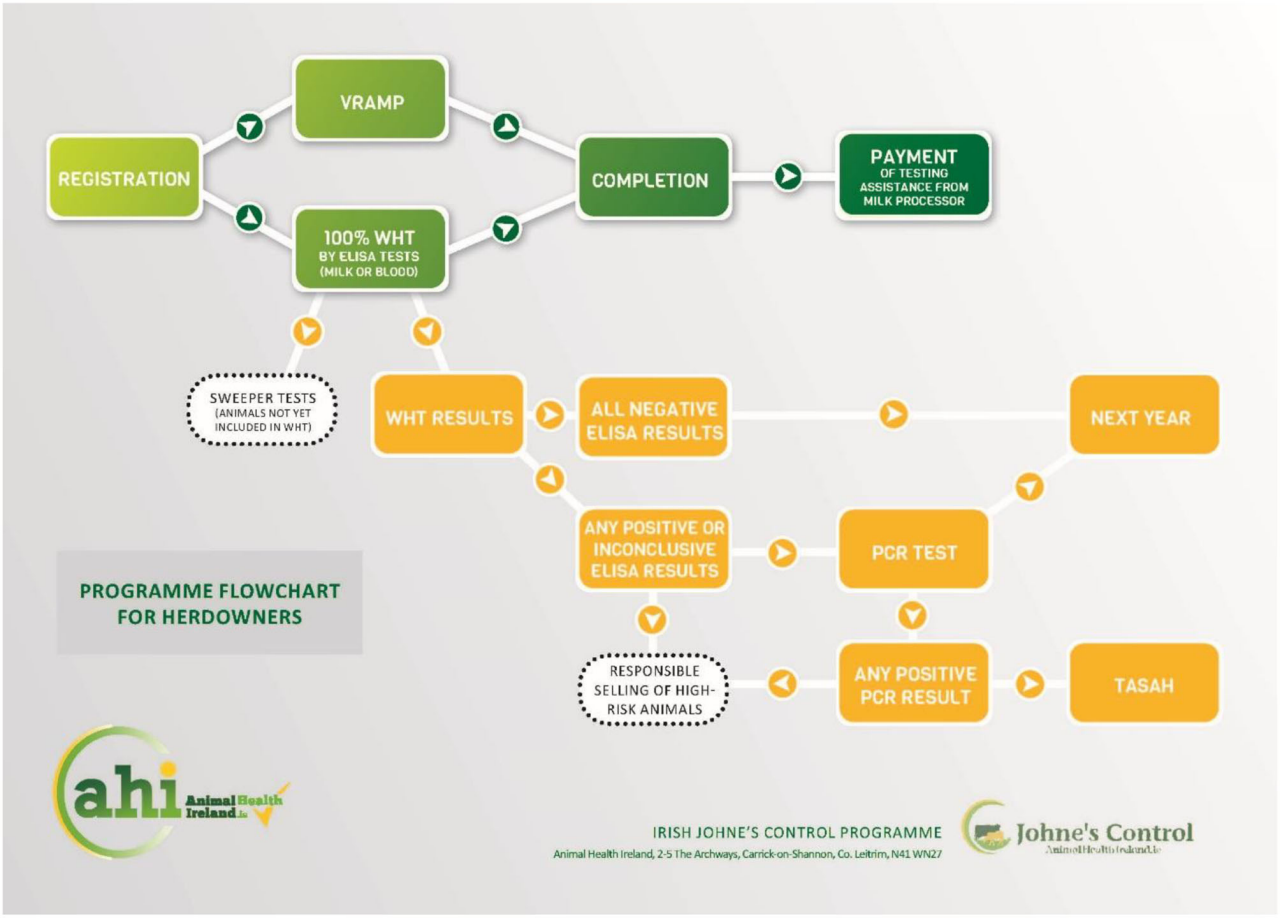
Programme flowchart (https://bit.ly/3hV8GHb).

JD testing and control can be complex, and a programme flowchart (Figure 3), available on the AHI website, displays the logical sequence of on-farm events, with embedded hyperlinks to details of how to complete each of the requirements of the IJCP.

However, in the first instance herdowners are encouraged to refer to their AVPs, who have undertaken training in all aspects of the IJCP, for technical advice and support. The funded activities also facilitate regular and closer engagement of veterinary services to support animal health and welfare generally.

AVPs are supported with a more technically detailed flowchart, and an exclusive web portal offering standard operating procedures, guidelines, templates, forms for laboratory submission and exempting animals from testing, and training materials. Although AVPs are invariably committed to their clients and the programme, most have only a small number of IJCP clients. Most commonly, AVPs have only a single herd in the programme, with a median of 3 per AVP, although a small number have considerably more.

Especially for those AVPs with small numbers of IJCP herds, allocating scarce time to maintaining and updating expertise may not be commercially viable. This expertise is essential to advising on the more nuanced and technical elements of the programme, such as interpretation of ELISA test results in the context of herd history, development of herd management plans, and epidemiological assessment of separate non-breeding units to exempt livestock from testing. However, their engagement in the programme does facilitate regular contact with clients, to foster the professional relationship and facilitate clinical and herd-health work.

The programme employs a range of engagement mechanisms, including: automated SMS messages from ICBF to herdowners upon upload of test results that suggest next steps and direct them to AVPs for more information; publication of regular bulletins to AVPs and to stakeholders including farmers; and webinars. Recorded videos and a podcast series were trialled with good initial effect, although audiences and engagement declined over time. A focus of these communications has been to provide simple and consistent messages.

A Facebook group has been successful at enabling AVPs to maintain their knowledge and constructively share their learnings and individual experiences with other AVPs, with no adverse comments requiring moderation; however, the active participants are those AVPs with multiple clients registered in the IJCP, so possibly is less frequently accessed by those AVPs with fewer dairy clients, possibly because the practise business model focuses on other activities. A similar Facebook group for herdowners is under consideration.

Programme communications in 2020 were curtailed by COVID-19 restrictions on face-to-face meetings and training. Formal meetings of the IG and the TWG continued on-line. The value of previously established good working relationships and familiarity with technology enabled the continuity of business in the face of otherwise challenging conditions. In contrast, initiating on-line meetings with unfamiliar groups (e.g., non-participating herdowners) did not find ready acceptance. Industry advisors reported “on-line fatigue,” especially for herdowners and AVPs with urgent and operational demands on their time.

### Beef Herds

The programme has recently been broadened to apply to beef herds. It is expected to appeal to herdowners of either pedigree herds or commercial beef breeding herds with confirmed or suspected clinical disease. Pedigree herds may benefit by using a standardised protocol for market assurance and by seeing an increased demand for low-risk bulls to the dairy sector.

Cost sharing for herd testing assistance and VRAMP funding for beef herds has not yet been determined.

### Learnings

Development and early implementation of the IJCP holds learnings that may assist others who are planning JD control programmes.

Strategic engagement of key stakeholders in agreeing objectives and sharing responsibility for decision-making and funding was critical. Policies and procedures must balance technical precision and pragmatism in the context of commercial dairy production.

Farmers value simple, consistent, relevant and timely communications. Veterinary practitioners operating busy private practises may benefit from training beyond the technical elements of Johne's control, to equip them for the role of herd health advisor and for managing conflicts of interest. The inclusion of behavioural science perspectives from the outset may have foreseen and resolved barriers to participation.

### Next Steps

Sustaining Johne's control programmes can be challenging (29). At the start of 2021, the 1750 dairy herds registered in the IJCP (11.3% of Irish dairy herds) are likely to comprise the more innovative and “early-adopter” farming leaders as evidenced by participation in farm discussion groups (Teagasc—The Agriculture and Food Development Authority, pers. comm.), active engagement with veterinary and allied services and herd size (average of 130 cows for IJCP herds, compared to average of 110 cows for Ireland).

Future recruitment is likely to require a different approach to messages and communication channels to convince more conservative farmers—the “late majority” and “laggard” groups for the diffusion of innovations [(30), cited in (25)]—of the value of Johne's control and to participate in the programme. This approach will refine current communication practices, informed by proposed research into psychosocial influences to engagement as described below.

The context of Johne's control is changing, as the Irish government promotes a National Farmed Animal Biosecurity Strategy that references JD (5) and milk processors promote milk recording that offers convenient and minimal-cost JD herd testing. Although currently performed in only 43% of herds, milk recording is being driven by national sustainability targets fostering improvements in herd productivity, milk quality goals, and emerging regulatory restrictions on use of antimicrobial therapeutics, including dry cow intra-mammary preparations, unless under veterinary prescription and with empirical evidence of aetiology and susceptibility.

A practical protocol for scoring herd risk, incorporating objective measures of risk from testing and histories of animal movements into each herd, and recognising VRAMP measures implemented to address individual farm mitigation priorities, is under development and expected to be released in 2021. This may provide additional incentives for farmers to register by providing tangible evidence of a herd's individual level of assurance. This could reward test-negative herds with voluntary marketing opportunities for low-risk breeding stock, encourage herdowners with infected herds towards effective biocontainment for their herds, and generally raise awareness of Johne's control.

A proposed behavioural science study will take an inductive approach to examine the experiences of participating farmers. By identifying motivations of participants and barriers and facilitators to completing yearly requirements, the study will support effective recruitment strategies to increase farmers' intrinsic motivation to join the programme, identify who is best to communicate key messages and provide recommendations to improve timely completion rates of annual WHTs and VRAMPs. It will also explore farmers' experiences of receiving test results and involve a collaborative co-design exercise to identify how best to communicate the complexities of the programme and the benefits of risk management recommendation uptake to end-users. This study will include determining whether the dissociation of annual cycles, between the programme based on the calendar year and farming practises based on seasonal events, is a significant deterrent. A second study will collate the experiences of AVPs with the aim of improving IJCP support to them.

AHI will continue to further incorporate JD control within broader biosecurity management, which was suggested by McAloon (24) as a means of ensuring a consistent approach to farm animal health risk management for a number of infectious animal health diseases which farmers manage routinely.

Work on developing metrics to determine progress in achieving programme objectives is to continue with the support and input from stakeholders.

## Conclusion

The IJCP has adopted a number of proven activities and new technologies to address the strategic perspectives of Irish dairy herds, viz the protection against infection for the estimated 70% of low-risk herds, and controlling the spread and impacts of JD for the estimated 30% of herds that are infected.

The most immediate challenges for sustaining and growing the IJCP are to maintain or improve the rate of recruitment of new herds, increase the completion rates for ancillary PCR testing and simplify the currently complex logistics of completing annual WHTs and VRAMPs within the seasonal cycles of milk production and bTB testing. The judgement of insufficient reward for the risk, inconvenience and expense of participation, especially for low-risk herds, remains a constant limitation and is recognised as an inhibitor to uptake of programmes internationally.

Information, tools and processes will continue to be refined, based on global and local scientific knowledge, to address the four agreed objectives of the programme.

## Data Availability Statement

The original contributions presented in the study are included in the article/supplementary material, further inquiries can be directed to the corresponding author/s.

## Author Contributions

LG and LC prepared the manuscript. DG and SM provided guidance and critical reviews. LG, DG, and SM are currently, and LC was formerly, members of the JD Implementation and the Technical Working Groups and have contributed to the development and implementation of the IJCP. All authors contributed to the article and approved the submitted version.

## Conflict of Interest

The authors declare that the research was conducted in the absence of any commercial or financial relationships that could be construed as a potential conflict of interest.

## Publisher's Note

All claims expressed in this article are solely those of the authors and do not necessarily represent those of their affiliated organizations, or those of the publisher, the editors and the reviewers. Any product that may be evaluated in this article, or claim that may be made by its manufacturer, is not guaranteed or endorsed by the publisher.
